# *Campylobacter jejuni* genes Cj1492c and Cj1507c are involved in host cell adhesion and invasion

**DOI:** 10.1186/s13099-020-00347-8

**Published:** 2020-02-11

**Authors:** De Xi, Thomas Alter, Ralf Einspanier, Soroush Sharbati, Greta Gölz

**Affiliations:** 1 Institute of Veterinary Biochemistry, Freie Universität Berlin, Berlin, Germany; 2 Institute of Food Safety and Food Hygiene, Freie Universität Berlin, Berlin, Germany

**Keywords:** *Campylobacter jejuni*, Mutants, Pathogenicity, Host cell response

## Abstract

**Background:**

*Campylobacter jejuni* (*C. jejuni*) has been assigned as an important food-borne pathogen for human health but many pathogenicity factors of *C. jejuni* and human host cell responses related to the infection have not yet been adequately clarified. This study aimed to determine further *C. jejuni* pathogenicity factors and virulence genes based on a random mutagenesis approach. A transposon mutant library of *C. jejuni* NCTC 11168 was constructed and the ability of individual mutants to adhere to and invade human intestinal epithelial cells was evaluated compared to the wild type. We identified two mutants of *C. jejuni* possessing altered phenotypes with transposon insertions in the genes Cj1492c and Cj1507c. Cj1492c is annotated as a two-component sensor and Cj1507c is described as a regulatory protein. However, functions of both mutated genes are not clarified so far.

**Results:**

In comparison to the wild type, Cj::1492c and Cj::1507c showed around 70–80% relative motility and Cj::1492c had around 3-times enhanced adhesion and invasion rates whereas Cj::1507c had significantly impaired adhesive and invasive capability. Moreover, Cj::1492c had a longer lag phase and slower growth rate while Cj::1507c showed similar growth compared to the wild type. Between 5 and 24 h post infection, more than 60% of the intracellular wild type *C. jejuni* were eliminated in HT-29/B6 cells, however, significantly fewer mutants were able to survive intracellularly. Nevertheless, no difference in host cell viability and induction of the pro-inflammatory chemokine IL-8 were determined between both mutants and the wild type.

**Conclusion:**

We conclude that genes regulated by Cj1507c have an impact on efficient adhesion, invasion and intracellular survival of *C. jejuni* in HT-29/B6 cells. Furthermore, potential signal sensing by Cj1492c seems to lead to limiting attachment and hence internalisation of *C. jejuni*. However, as the intracellular survival capacities are reduced, we suggest that signal sensing by Cj1492c impacts several processes related to pathogenicity of *C. jejuni*.

## Background

*Campylobacter* spp. are microaerophilic, gram-negative and motile bacteria belonging to the family of *Campylobacteraceae* [1]. *C. jejuni* is the most frequent cause of bacterial food borne disease worldwide [2–4]. The most common symptoms caused by *C. jejuni* are gastroenteritis usually accompanied by fever, vomiting, abdominal pain and bloody diarrhoea [1, 5]. In contrast to other enteropathogens, our understanding of pathogenetic mechanisms of *C. jejuni* lags behind and functions of many genes of *C. jejuni* still remain to be elucidated.

In the course of colonisation, *C. jejuni* can cross the mucosal barrier, adhere to as well as invade host epithelial cells, and produce one or more cytotoxins [3, 6]. Extensive studies have investigated that *C. jejuni* possesses numerous pathogenicity-associated factors involved in bacterial colonisation, transmigration and intracellular survival in intestinal epithelial cells [7, 8]. Two major known transmigration mechanisms are the transcellular and the paracellular pathway [8]. The transcellular route allows *C. jejuni* to cross the epithelial layer by attaching and invading at their apical surface, followed by escaping at the basolateral membrane. The paracellular pathway is characterised by breaking the tight junction and adherens junction complexes and crossing epithelial barriers by passage between neighbouring cells [7]. It has been demonstrated recently, that the *C. jejuni* protease HtrA is involved in breaching the epithelial barrier and enables invasion of epithelial cells via the paracellular pathway [9].

Hence, adhesion and invasion are recognised as important features in *C. jejuni* pathogenesis [10, 11]. There are several adhesion and invasion related genes already characterised in many in vivo and in vitro studies. The *flaA* and *flaB* genes, encoding major flagellin proteins, are strongly associated with invasiveness of *C. jejuni* [1]. Other genes such as *cadF* have been also implicated in adhesion. It is involved in the synthesis of a fibronectin-binding outer membrane protein facilitating attachment to glycoproteins of epithelial cells [12]. Mutagenesis based experiments indicate that several other genes, for example *ciaB*, *capA* and *flgB* are involved in binding and invasion of host intestinal epithelial cells [1, 5].

*Campylobacter jejuni* infection can induce the expression of various cytokines [13–15]. Interleukin-8 (IL-8) is an important pro-inflammatory chemokine of intestinal epithelial cells and acts as a chemotactic factor of immune cells. It can be induced during infection by adhesion and/or invasion as well as exposure to the cytolethal-distending-toxin (CDT) produced by *C. jejuni* [13, 14, 16, 17]. While research in the past decade has focused on mechanisms of *C. jejuni* interaction with host cells, the knowledge on its intracellular fate is still limited. Only few genes have been reported to be involved in intracellular survival of *C. jejuni* [18].

Studies have proved that the intestinal mucosa behaves as the first barrier against microbial infections. Since human intestinal epithelial cells are polarised and possess a luminal mucous layer [7], adhesion, invasion and translocation ability of *C. jejuni* have been shown to depend on these characteristics. In our study, we have used the epithelial cell line HT-29/B6, a sub clone of HT-29 cell line, which has been shown to produce a mucus layer [19] providing an appropriate model for *C. jejuni* interaction.

In this study, we applied an approach utilising in vitro integration of a transposon in *C. jejuni* genomic DNA followed by natural transformation [20, 21]. A transposon mutant library was constructed in *C. jejuni* NCTC 11168, from which two mutants with altered capability to invade human intestinal epithelial cells have been selected and used for further phenotypical characterisation. The aim of the present study was to discover hitherto unknown pathogenicity factors of *C. jejuni* with respect to adhesion, invasion, intracellular survival and host cell response. We found that mutagenesis of Cj1492c and Cj1507c, two so far poorly characterised genes of *C. jejuni* NCTC 11168, has remarkable effects on adhesion and invasion of host cells in a reverse manner. However, they seem not to influence host cell pro-inflammatory responses.

## Results

### Transposon mutagenesis of *C. jejuni* NCTC11168

**Fig. 1:**
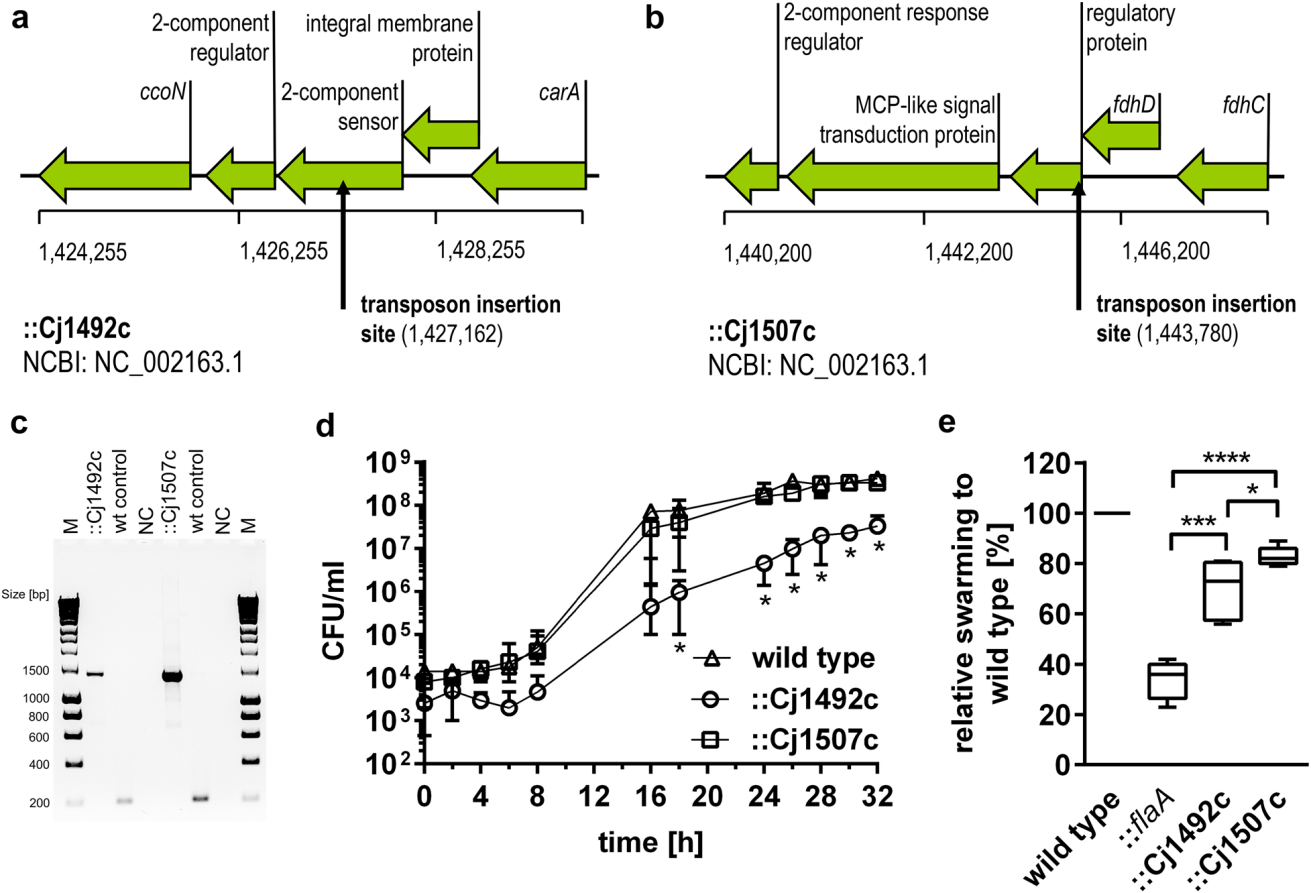
Genomic locus, transposon insertion sites in *C. jejuni* NCTC 11168 mutants ::Cj1492c and ::Cj1507c and PCR verification of both mutants and phenotypic analyses of two mutants. **a**, **b** Genes are plotted as arrows in order to their genomic locations. Boxed arrows length indicates relative gene sizes. **a** The location of inserted transposon is indicated in *C. jejuni* two-component sensor gene (Cj1492c). **b** The location of the inserted transposon is indicated in *C. jejuni* regulatory protein gene (Cj1507c). **c** Agarose gel electrophoresis of genomic DNA of mutants ::Cj1492c, ::Cj1507c and wild type using gene specific primers. With integration of the transposon (1219 base pairs) the DNA fragment sizes of both mutants are around 1400 base pairs in length which are 1.2 kb larger than the target DNA of the wild type (around 200 base pairs). **d** Growth curve of wild type, ::Cj1492c and ::Cj1507c were assessed by CFU counting. Values are shown as mean value of three independent experiments. Error bars represent SD. Statistical comparison with the wild type: *p ≤ 0.05, unpaired *t*-test. **e** Differential swarming ability of mutated strains was normalised with the wild type strain and is presented as percentage of swarming halos of wild type (100%). Columns show median value of five biological replicates and bars indicate maximum and minimum range. *p ≤ 0.05; ***p ≤ 0.001; ****p ≤ 0.0001, unpaired *t*-test

In this study, the HyperMu <KAN-1> Transposon was randomly inserted into the DNA of *C. jejuni* NCTC 11168 using a 2 h in vitro reaction catalysed by HyperMu MuA Transposase and subsequently transformed into *C. jejuni* NCTC 11168 by natural competence. This in vivo protocol successfully generated 24 kanamycin-resistant mutants in *C. jejuni* NCTC11168. Transposon insertion site was determined by sequencing with the primers included in the HyperMu <KAN> Transposon Kit and verified by gene specific PCR amplification. The sequence data proved that each mutant had a single transposon insertion and transposons were inserted randomly on *C. jejuni* chromosome (data not shown). To identify genes involved in *C. jejuni* pathogenicity, the obtained mutants were subsequently screened using invasion assays with HT-29/B6 cells. Two mutants (::Cj1492c and ::Cj1507c) that showed differing invasiveness compared to the wild type were chosen to assess detailed phenotypic studies. However, the function of mutated genes is not clarified so far. One insertion is located in the gene Cj1492c, annotated as a two-component sensor and the second one is located in the gene Cj1507c, described as a regulatory protein, involved in molybdenum and tungsten transport [22]. The maps of Cj1492c and Cj1507c genes and transposon insertion sites are shown in Fig. 1a, b. The results of a gene specific PCR are shown in Fig. 1c. Both mutants possessed an approximately 1.2 kb larger fragment compared to the parental target DNA indicating that these genes contained a transposon insertion.

### Growth dynamics

Both mutants (::Cj1492c and ::Cj1507c) and the wild type were separately inoculated in Brucella broth (BB) and incubated for 48 h at 37 ℃ under microaerobic conditions and viable cell counts were determined over time. A growth curve including lag (0–8 h), exponential (8–18 h) and stationary (18–32 h) phases is presented in Fig. 1d. Strain ::Cj1507c grew almost identically to the wild type whereas ::Cj1492c showed apparent differences in growth dynamics. Early growth rate of ::Cj1492c was clearly retarded compared to the wild type as represented by a flatter slope of the growth curve between 6 and 18 h and approx. 100-fold lower number of viable bacteria during exponential phase. After 28 h, ::Cj1492c reached a stationary phase with significantly diminished number of viable bacteria compared to the wild type and ::Cj1507c and there was no difference between the two other strains.

### Swarming ability of ::Cj1492c and ::Cj1507c

As motility is essential for host invasion [1, 18], ::Cj1492c, ::Cj1507c, wild type and a ::*flaA* mutant (used as a negative control) were tested on semisolid Mueller–Hinton agar (MH) plates to examine whether *C. jejuni* swarming ability is affected in the mutated strains. Swarming ability of the three mutants was normalised to the wild type. As expected, ::*flaA* was found to be barely motile (36%), which is consistent with previous studies [1, 23–25]. In contrast and as shown in Fig. 1e, both mutant ::Cj1492c and ::Cj1507c, showed slightly reduced swarming ability (approx. 73% and 82% of wild type swarming in MH, respectively).

### Adhesion and invasion of human intestinal epithelial cells by ::Cj1492c and ::Cj1507c

**Fig. 2:**
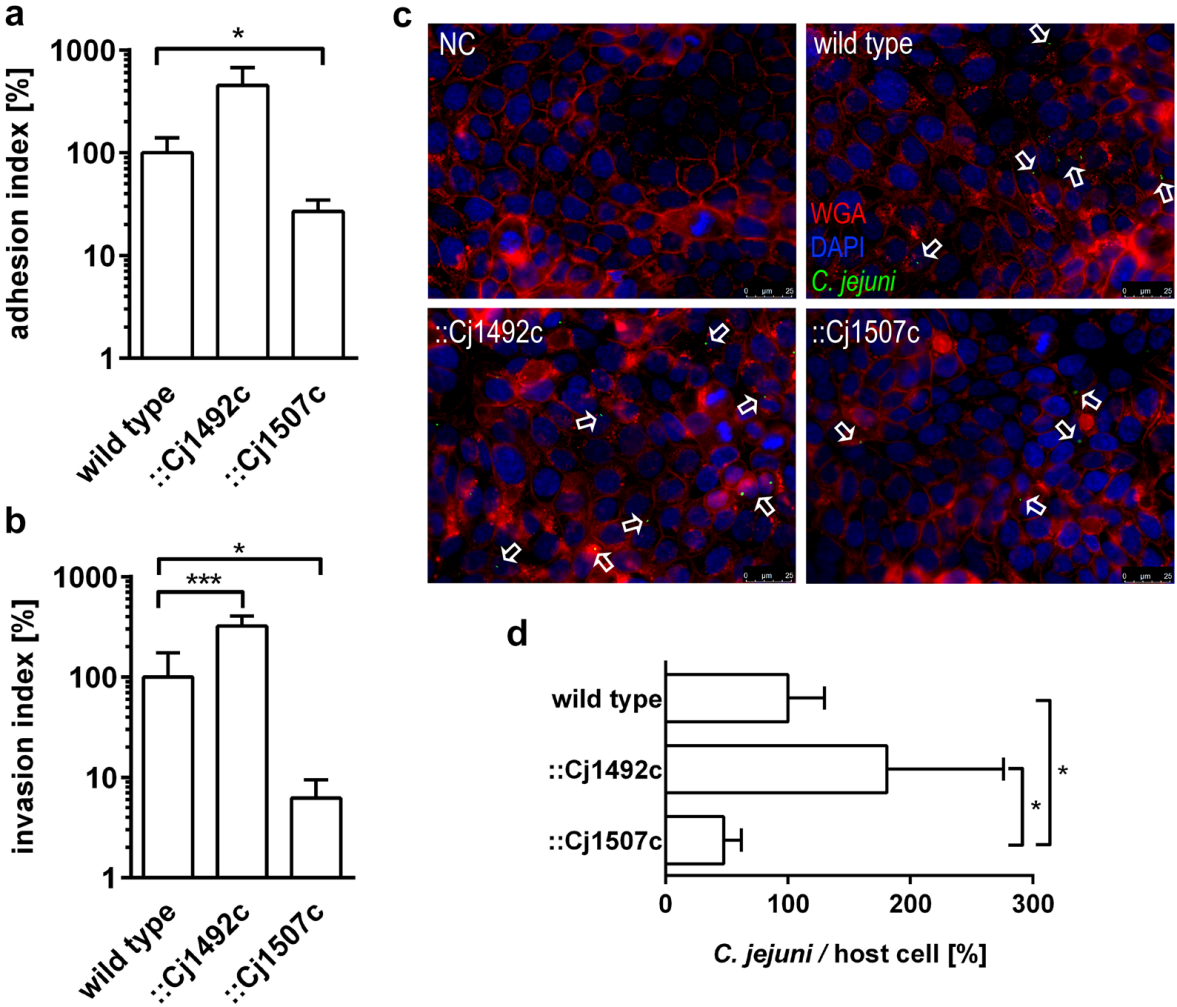
Adhesion, invasion and internalisation levels of *C. jejuni* in HT-29/B6 cells and immunofluorescent detection of intracellular *C. jejuni*. Adhesion (**a**) of three tested strains to HT-29/B6 was detected at 1 h p.i.. For determination of invasion (**b**), the monolayer of HT-29/B6 was incubated with gentamicin for an additional 2 h prior to lysis. Experiments were performed at least three independent times in triplicate. The relative adhesion and invasion index were normalised with wild type and are presented as percentage of the adhesion and invasion index of wild type, respectively. Results show the mean value ± SD. *p ≤ 0.05; ***p ≤ 0.001, unpaired *t*-test. **c** HT-29/B6 cells and immunofluorescently labelled wild type, ::Cj1492c and ::Cj1507c (green, indicated by white arrows) and untreated control cells are shown at 5 h p.i. Cell cytoplasm membrane were stained with WGA-CF^®^594 (red) and nuclei with DAPI (blue). Scale bars indicates 25 μm. **d** The internalisation level of *C. jejuni* by HT-29/B6 was determined as a ratio of particles of invaded *C. jejuni* and infected cells by means of immunofluorescence microscopy and automatic particle counting using ImageJ. The relative level of internalisation was normalised with wild type and is presented as percentage of the wild type. Fluorescent particles of each strain were quantified in at least six microphotographs selected randomly for each biological replication and results are presented for four individual experiments. Asterisks show statistically significant differences between ::Cj1492c and ::Cj1507c. *p ≤ 0.05; unpaired *t*-test

Both mutants along with the wild type were tested for their abilities to adhere to and invade HT-29/B6 host cells. Therefore, HT-29/B6 monolayers (with approx. 80–90% confluence) were infected with approx. 1 × 10^9^ colony-forming units (CFU) of each strain. The strain ::Cj1492c showed more than threefold but statistically not significantly enhanced adhesion to HT-29/B6 cells compared to the wild type (0.037% of ::Cj1492c vs. 0.011% of wild type inoculum adhered to host cells). However, disruption of Cj1507c caused clearly and significantly (p ≤ 0.05) decreased adhesion to host cells. Only 0.003% of ::Cj1507c inoculum adhered to HT-29/B6, which corresponds to 27% of the wild type (Fig. 2a).

In accordance with the adhesion analysis, we determined that mutant ::Cj1492c invaded epithelial cells approx. 3-times more efficiently than the wild type (0.0049% of inoculum for ::Cj1492c vs. 0.0015% for wild type, p ≤ 0.001), while the strain ::Cj1507c showed tenfold reduced (p ≤ 0.05) ability to invade HT-29/B6 cells compared to the wild type (Fig. 2b).

### Immunodetection of intracellular *C. jejuni*

With regard to adhesion and invasion, we examined that mutation of the genes Cj1492c and Cj1507 causes contrary phenotypes. In order to determine the internalisation level of *C. jejuni* in HT-29/B6, immunofluorescence staining along with WGA and DAPI staining were performed up to 5 h post infection (p.i.). WGA is a carbohydrate-binding lectin that has high affinity for sialic acid and *N*-acetylglucosamine [26] and is used as a marker for the cytoplasm membrane of host cells. To subtract unspecific immunofluorescent signal, non-infected HT-29/B6 cells, which were stained the same as the infected samples, were used as a negative control. There was no immunofluorescent signal detected in the negative controls. As representatively shown in Fig. 2c, several bacteria seemed to be located at the intercellular spaces. No specific differences were found among the tested strains. Furthermore, we quantified the number of invaded bacteria per host cell at 5 h p.i. by means of fluorescent particle counting using ImageJ [27, 28]. The average number of wild type *C. jejuni* per host cell was ~ 0.08 bacteria/host cell. As shown in Fig. 2d, the mutant ::Cj1492c exhibited increased levels of internalisation compared to the wild type (181% of the wild type) but was not statistically significant, whereas ::Cj1507c possessed half the internalisation of the wild type (47% of the wild type, p  ≤ 0.05). These data are in line with our observations in the invasion assay shown above.

### Intracellular persistence of the mutants ::Cj1492c and ::Cj1507c

**Fig. 3:**
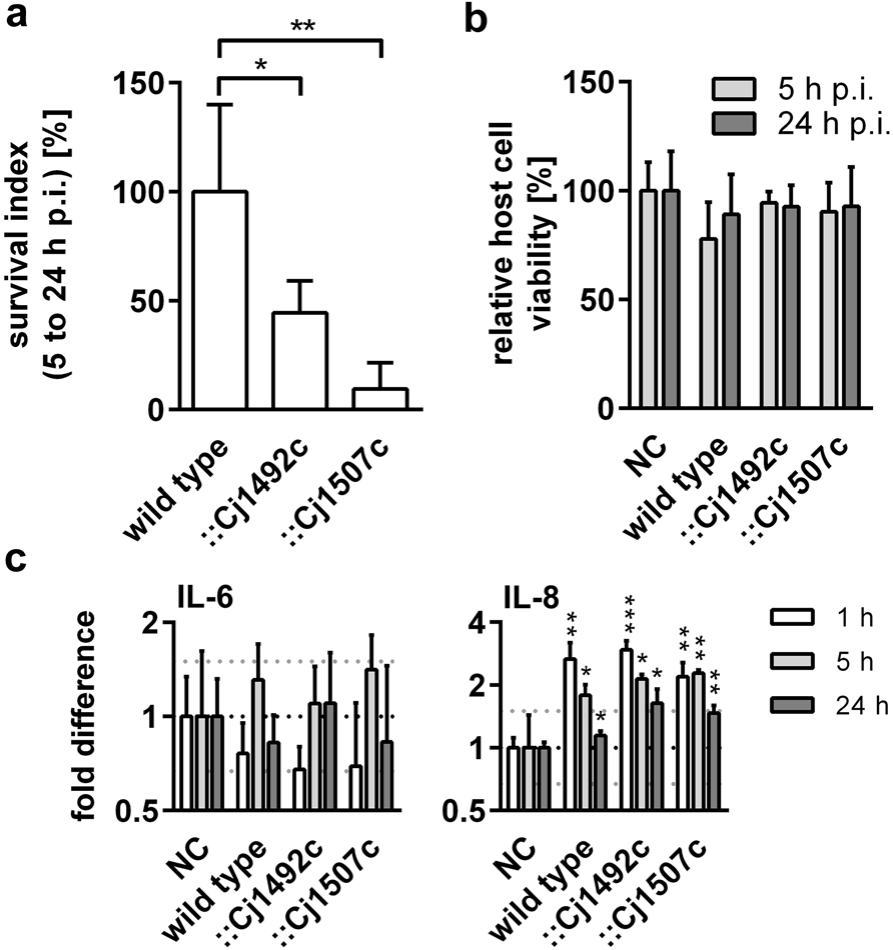
Intracellular survival of *C. jejuni*, relative viability of HT-29/B6 cells and RT-qPCR analysis of IL-6 and IL-8 mRNA expression in HT-29/B6 cells after *C. jejuni* infection. **a** Intracellular survival, presented as CFU recovery rates of intracellular viable cell counts, was determined as a ratio of viable counts between 5 and 24 h p.i. The relative survival rate was normalised with the wild type and is presented as percentage of the wild type. Results represent at least six individual experiments and asterisks indicate statistically significant differences between mutants and the wild type. *p ≤ 0.05; **p ≤ 0.01, unpaired *t*-test. **b** Relative viability of HT-29/B6 cells after infection was measured after 5 h and 24 h of infection and calculated as ratio of number of viable cells compared to the total cell numbers and normalised on the viability of uninfected control group. Values are the mean ± SD of three individual experiments. **c** Fold changes of IL-6 and IL-8 mRNA expression were calculated relatively to non-infected controls and normalized to housekeeper. Experiments were performed in triplicate and the mean ± SD is presented. Only altered mRNA expression level above or below the dotted lines are defined as regulated. Asterisks indicate statistically significant differences between tested strains and negative controls at each time point. *p ≤ 0.05; **p ≤ 0.01; ***p≤ 0.001, unpaired *t*-test

We were able to show that these two mutants possess adhesion and invasion capabilities in a reverse manner but retain only slightly diminished swarming abilities compared to the wild type. Furthermore, we wanted to address whether both mutants showed altered abilities to persist intracellularly. Therefore, the number of intracellular bacteria was determined over time by examining the CFU at 5 and 24 h p.i., respectively. More than two-thirds of the intracellular wild type *C. jejuni* were eliminated between 5 and 24 h p.i. while approx. 85% of intracellular ::Cj1492c and more than 95% of the intracellular ::Cj1507c were eliminated within this time. As indicated in Fig. 3a, the mutant ::Cj1492c exhibited half the survival rate of the wild type (48.1% of wild type, p ≤ 0.05) and the intracellular persistence of strain ::Cj1507c was only one-tenth of the wild type (9.4% of wild type, p ≤ 0.01). Both mutants showed significantly diminished intracellular survival rates compared to the wild type. However, there was no statistically significant difference between the mutants. Regardless of the mutation, these data showed that most intracellular *C. jejuni* were killed within the initial 24 h p.i. in HT-29/B6.

### Effects of mutations on host cell viability

For determining effects of *C. jejuni* strains on host cell survival, we tested the viability of HT-29/B6 cells at 5 and 24 h p.i. based on Calcein-AM/Hoechst staining [29, 30]. This assay quantitatively determines the number of viable host cells indicated by the Calcein signal versus the total counts of cells represented by Hoechst staining. To assess relative host cell survival after infection, the ratio of viable to total cell numbers was calculated in relation to non-infected cells. As shown in Fig. 3b, there was no significant difference in host cell viability among tested stains and for the duration of the experiment over 24 h.

### Cytokine induction by *C. jejuni* mutants in HT-29/B6 cells

To assess the effects of mutations on the induction of pro-inflammatory cytokines, the mRNA expression of IL-8 and IL-6 was evaluated by calculating the relative fold difference to non-infected cells (controls). The results (Fig. 3c) indicated that all strains uniformly induce IL-8 mRNA expression of host cells at 1 h p.i. followed by a decrease until 24 h p.i. in a similar manner. Although individual mRNA patterns of IL-6 have been observed during the infection of all strains, no significant differences were observed.

## Discussions

Growing evidence has shown that adhesion and invasion is mediated by multiple factors that are critical for pathogenesis of *Campylobacter* [1, 4, 5, 11]. However, the molecular mechanisms of pathogenicity are not fully understood. To continue identifying unknown virulence genes, a transposon mutant library of *C. jejuni* NCTC 11168 was constructed and the ability of individual mutants to adhere to and invade human intestinal epithelial cells was evaluated compared to the wild type. We identified two mutants of *C. jejuni* possessing altered phenotypes with transposon insertions in the genes Cj1492c and Cj1507c.

Gene Cj1492c, annotated as a two-component sensor belongs to the class of Per-Arnt-Sim motif (PAS) domain-containing sensor histidine kinases, for which binding of a chemically diverse range of small-molecule metabolites is typical [31, 32]. It has been reported that mutagenesis of gene Cjj1484 (the orthologue of Cj1492c in *C. jejuni* 81–176) was accompanied with the repression of several genes involved in metabolism, iron/heme acquisition and respiration during cultivation in broth, while no influence on colonisation of chickens was determined [33]. Others have reported that the expression of Cj1492c was up-regulated after treatment with 0.1% sodium deoxycholate and down-regulated after treatment with epinephrine and norepinephrine [34, 35]. However, neither the specific signalling molecule nor the function have yet been clarified.

The gene Cj1507c is annotated as a regulatory protein belonging to the family of LysR transcriptional regulators [22]. But little is known about the functions of LysR family regulators in *Campylobacter*. Another LysR regulator (Cj1000) is reported to facilitate adaption of *C. jejuni* to host colonisation and respiration [36]. Taveirne et al. [22] described that Cj1507c is involved in the repression of the molybdenum transport system *modABC* as well as the tungsten transport system *tunABC* and therefore termed as *modE* [22]. In contrast to other ModE proteins, the *C. jejuni* ModE does not contain a metal binding domain but instead a protein–protein binding site. This indicates that ModE could be a DNA-binding subunit of a multiprotein complex involved in the repression of both transport system operons [22]. Additionally, it has been reported that molybdenum plays an important role during intestinal colonisation of mice by *C. jejuni* 81-176 [37].

Previous studies determined that *C. jejuni* motility is necessary for colonising the mucous layer of the gastrointestinal tract and therefore is important for *C. jejuni* invasion of epithelial cells [1, 4, 5, 8, 18, 38]. The roles of motility, adhesion, invasion or their mutual interplay during pathogenesis have been partially clarified. It is worth noting that both mutants ::Cj1492c and ::Cj1507c retain slightly reduced motility but behave in a reverse manner in terms of adhesion and invasion. While mutagenesis of Cj1507c caused a severe reduction in adhesion and invasion, the disruption of Cj1492c contributed to a significantly enhanced adhesion and invasion. Therefore, it seems unlikely that the slightly reduced motility is the reason for the divergent effects observed for both mutants.

Furthermore, genes involved in intracellular survival have been reported to be also involved in invasion [18, 39]. In order to detect whether mutations of genes Cj1492c and Cj1507c caused altered intracellular survival, we determined the CFU of intracellular *C. jejuni* after 5 and 24 h p.i.. In our experiments, the amount of culturable intracellular bacteria was severely decreased after 24 h for both mutants and wild type. An accentuated difference (one-tenth) in the survival rate between ::Cj1507c and the wild type was found and ::Cj1492c showed intermediate survival (one-half) compared to the wild type. Survival mechanisms of *C. jejuni* in eukaryotic cells are still not fully understood. It has been reported that *C. jejuni* undergoes intracellular metabolic reprogramming upon internalisation contributing to subsequently impaired intracellular persistence [40, 41]. Hence, we assume that both genes Cj1492c and Cj1507c are also involved in the physiological adaptation of *C. jejuni* to the hostile intracellular environment.

Numerous cytotoxins produced by *C. jejuni* might be engaged in infection of host cells. Therefore, we addressed potential correlations between adhesion and invasion along with the impact of tested strains on host cell survival in HT-29/B6 cells after infection. In fact, we observed that there was a small but non-significant decline in the number of viable host cells between infected and non-infected samples. However, no difference was found among wild type and two mutants neither at 5 nor at 24 h p.i.. As the wild type did not impair host cell viability in the time course of our experiments, which might be due to the short period of infection, we cannot exclude potential effects of mutations on host cell cytotoxicity upon infection at later time points.

Similarly, to what had been previously published, we observed significantly enhanced levels of IL-8 mRNA in all infected samples compared to the non-infected controls [42–44]. After an early peak, there was a decrease in expression of IL-8, which agrees with previous observations regarding a down-regulation of pro-inflammatory cytokines after early peak to protect epithelial cells from chronic inflammation and epithelial destruction [11]. We found that IL-8 expression in response to infection was similar among tested strains throughout the infection course. These data suggest that genes Cj1492c and Cj1507c are not responsible for the induction of IL-8 expression in host cells. Furthermore, the IL-8 mRNA induction in our experiments appears to be independent from capabilities to adhere and to invade host cells or the intracellular persistence. No significant regulation of IL-6 mRNA expression was induced by both mutants and wild type along the entire infection course. According to a previous study, IL-8 is important for extracellular response, whereas IL-6 is considered as an important factor for integrity of epithelial cells and plays a vital role on the intracellular response [45].

The strategies of *C. jejuni* to invade epithelial cells are known as paracellular- and transcellular-pathways [7, 8]. In our immunofluorescent detection, several *C. jejuni* seemed to be located in the intercellular spaces. This might indicate that *C. jejuni* paracellularly transmigrate via cell junctions and then invade the epithelial cells at the basolateral membrane, since the CadF and FlpA binding-protein fibronectin is predominantly located on the basal site of enterocytes [9]. However, by the method applied in our study, we could not exactly distinguish whether the transmigration of tested strains and the infection of HT-29/B6 cells occurred via the trans- or paracellular pathway.

The observed phenotypical differences of mutants might also result from polar effects of the mutation. For the downstream located cognate response regulator of the two-component sensor Cj1492c, it has been reported that it influences expression of some specific genes independently of the histidine kinase Cjj1484 [33]. The gene upstream of Cj1492c encodes an integral membrane protein, containing a conserved protein domain of the possible sulfite exporter TauE/SafE family, which has not been well characterised in *C. jejuni* thus far. The downstream gene of Cj1507c encodes the chemoreceptor Tlp1, responsible for sensing aspartate [46], and mutation of *tlp1* resulted in enhanced adhesion and invasion of CaCo2 cells [47]. The gene *fdhD*, located upstream of Cj1507c, encodes an accessory protein of the formate dehydrogenase, which also has not been well investigated in *C. jejuni*. However, it has been demonstrated that mutation of *fdhD* resulted in similar colonisation abilities but reduced immunopathological responses in mice [48]. Nevertheless, it still remains unclear whether potential polar effects generated by mutagenesis of Cj1492c and Cj1507c play a role or not.

## Conclusion

We conclude that genes regulated by Cj1507c have an impact on efficient adhesion, invasion and intracellular survival of *C. jejuni* in HT-29/B6 cells. Furthermore, signal sensing by Cj1492c seems to lead to limiting attachment and hence internalisation of *C. jejuni*. However, as the intracellular survival capacities are reduced, we suggest that signal sensing by Cj1492c may impact several processes related to pathogenicity of *C. jejuni* in HT-29/B6 cells. A more complete understanding of the function of Cj1492c and Cj1507c as well as the regulatory networks is subject of our future investigations.

## Methods

### Bacterial strains and culture conditions

All bacterial strains used in this study (*C. jejuni* NCTC 11168 and mutants ::Cj1492c, ::Cj1507c and ::*flaA*) were routinely grown on Mueller–Hinton agar (Oxoid, Munich, Germany) supplemented with 5% defibrinated sheep blood (MHA) or in Brucella broth (BB; BD, Heidelberg, Germany) at 37 ℃ under microaerobic conditions (10% CO_2_, 6% O_2_ and 85% N_2_) generated by an Anoxomat (Omni Life Science, Bremen, Germany) as described previously [49]. For invasion, adhesion and infection assays, *C. jejuni* strains were grown to mid-exponential phase (approx. 20 h) in BB at 37 °C under microaerobic conditions and harvested by centrifugation (14,000*g*, 5 min). Cell pellets were resuspended in appropriate buffers or media for further experiments. All in vitro infections with bacteria were performed at a multiplicity of infection (MOI) of 500.

### Random transposon mutagenesis

*Campylobacter jejuni* NCTC 11168 (wild type) was incubated for 24 h in BB at 37 ℃ under microaerobic conditions. DNA was extracted from this culture with Easy-DNA-Extraction kit (Invitrogen, Thermo Fisher Scientific, Waltham, US) according to manufacturer’s instructions. The extracted DNA was used for further insertion of HyperMu <KAN1> transposon (Epicentre, Madison, US). Briefly, a total volume of 40 µl containing 5 µg DNA, 1× reaction buffer, 50 ng Transposon and 2 U Transposase were incubated for 2 h at 37 ℃. Reaction was stopped by adding the stop solution and incubation at 70 ℃ for 10 min and was stored at − 20 ℃ for further use. Wild type was mutated by natural transformation. In brief, *C. jejuni* NCTC 11168 was incubated in BB for 24 h at 37 ℃ under microaerobic conditions and diluted to OD_600_ = 0.1. MHA in glass tubes was overlaid with 0.5 ml of this culture and incubated for 3 h. After addition of 5 µg genomic DNA with integrated transposons, the culture was further incubated for 20 h at 37 ℃ under microaerobic conditions. This culture was streaked on MHA plates containing 20 µg/ml Kanamycin (Sigma-Aldrich, St. Louis, MO, USA) and incubated for 72 h to select transformants. Each grown colony was enriched by 48 h incubation on MHA containing 50 µg/ml Kanamycin. Insertion site was determined by sequencing with the primers included in the HyperMu <KAN> Transposon Kit and further verified by PCR amplification with gene specific primers. All primers used in this study are listed in Table 1.

**Table 1:** Primers used in this study

Gene	Primer	Sequence
Cj1492c	Cj1492c_fw	TTT GAT TGT GAG TAA ATT TCA GCA A
	Cj1492c_rev	ATG CAA ATT GCC TTG GAA AA
Cj1507c	Cj1507c_fw	TCG GCT AAT TGT CCG ATT TT
	Cj1507c_rev	CGC TTT GTG GTT TTG CTA GA
*flaA*	flaA_fw	ACG ATA TAG CAT TTA ACA AG
	flaA_rev	AAC AAC TGA ATT TGC ATG TGC
Transposon	MUKAN-1 FP-1 forward primer	CTG GTC CAC CTA CAA CAA AGG
	MUKAN-1 RP-1 reverse primer	AGA GAT TTT GAG ACA GGA TCC G
IL-6	hsa_IL6_fw	GAA AGC AGC AAA GAG GCA CT
	hsa_IL6_rev	TTT TCA CCA GGC AAG TCT CC
IL-8	hsa_IL8_fw	GTG CAG TTT TGC CAA GGA GT
	hsa_IL8_rev	CTC TGC ACC CAG TTT TCC TT
ACTB	hsa_ACTB_fw	GGA CTT CGA GCA AGA GAT GG
	hsa_ACTB_rev	AGC ACT GTG TTG GCG TAC AG
B2M	hsa_B2M_fw	GTG CTC GCG CTA CTC TCT CT
	hsa_B2M_rev	GGA TGG ATG AAA CCC AGA CA

Primers used for partial amplification were designed with Primer3 version 0.4.0 [50] based on whole-genome sequence of *C. jejuni* NCTC 11168 [32]. To verify the mutated genes 0.4 µM of each primer, 1× PCR buffer (Qiagen, Hilden, Germany), 1.5 mM MgCl_2_ (Qiagen), 0.4 mM of each dNTPs (Fermentas, St. Leon-Rot, Germany), 0.5 U Taq-Polymerase (Qiagen) and 2 µl extracted genomic DNA of each mutant were used in PCR. The following amplification protocol was used: initial denaturation for 5 min at 95 ℃ followed by 35 cycles with 30 s at 94 ℃, 60 s at 59 ℃ and 60 s at 72 ℃. Afterwards, a final elongation step was performed for 7 min at 72 ℃. PCR amplification products were separated by 1.5% agarose gel-electrophoresis and fragment size was determined according to the Hyperladder 1 kb Plus (Bioline, Luckenwalde, Germany) standards.

### Growth curve

Growth curves of wild type, ::Cj1492c and ::Cj1507c were generated as previously described with few modifications [49]. Briefly, 5 ml BB was inoculated with freshly grown bacteria of each strain and incubated overnight (approximately 18 h) at 37 ℃ under microaerobic conditions. These pre-cultures were diluted in BB to reach a starting culture with approximately 6 × 10^3^ CFU/ml and incubated at 37 ℃ for 48 h under microaerobic conditions. Viable bacterial numbers were determined at indicated time points by plating serial dilutions (10^−1^) on MHA plates. Before determining the CFU, plates were incubated at 37 ℃ under microaerobic conditions for 48 h. Experiments were repeated independently at least three times and CFU/ml was determined in duplicate.

### Swarming assay

Swarming assays were performed according to previous studies [6, 49] with some modifications. The wild type and the mutant strains (::*flaA*, ::Cj1492c, ::Cj1507c) were cultured in BB for 20 h at 37 °C under microaerobic conditions and adjusted to 1 × 10^8^ CFU/ml. 1 μl of each overnight culture was stabbed into Mueller–Hinton plates containing 0.4% agar (MH) and incubated microaerobically at 37 ℃ for 24 h. To minimise plate-to-plate variations, all tested mutant strains and the wild type were included on the same agar plate. The diameter of formed halo of the mutants was measured and normalised to the wild type from the same plate. Each strain was analysed performing at least five independent assays.

### Cultivation of human epithelial cells

The sub clone HT-29/B6 [19] of the human colorectal adenocarcinoma cell line HT-29 (DSMZ_ACC 299) was routinely cultured as described previously [51]. Briefly, HT-29/B6 cells were cultured in RPMI 1640 medium (Lonza, Basel, Switzerland) supplemented with 10% (v/v) FCS superior (Biochrom, Cambridge, United Kingdom) in 75 cm^2^ tissue culture flasks (Sarstedt, Nümbrecht, Germany) at 37 ℃ and 5% CO_2_ under a humidified atmosphere until a confluence of approx. 80–90% was reached. For adhesion and invasion assays, 1 × 10^5^ HT-29/B6 cells were seeded into each well of a 24 well plate (Sarstedt) and incubated for 7 days changing the media every 3 days. For examining cytotoxicity, each well of a 96 well plate (Sarstedt) was seeded with 1.8 × 10^4^ HT-29/B6 cells and incubated as described above. For gene expression analysis, infection assays were performed in 6 well plates (Sarstedt) with 5 × 10^5^ HT-29/B6 cells seeded per well and following incubation at 37 ℃ and 5% CO_2_ routinely changing the medium for 7 days.

### In vitro adhesion and invasion assay

Adhesion and invasion assays with the cell line HT-29/B6 were performed with wild type, ::Cj1492c and ::Cj1507c as previously described with slight modification [51, 52]. Briefly, approximately 1 × 10^5^ HT-29/B6 cells were seeded into each well of a 24 well plate. After 7 days of differentiation, cells with ~ 80–90% confluence were infected with a suspension of approximately 1 × 10^9^ CFU of bacteria (MOI 500) for both adhesion and invasion assays. To determine the adhesion of each strain, monolayers were infected for 1 h and incubated at 37 ℃ with 5% CO_2_. Afterwards, infected cells were rinsed with phosphate buffered saline (PBS, Sigma-Aldrich) to wash off loosely attached *C. jejuni* and then lysed with 1% Triton-X-100 (Carl Roth, Karlsruhe, Germany) for 10 min at room temperature. For invasion assays, infected monolayers were incubated for 3 h and subsequently treated with 300 ng/ml gentamicin for 2 h (Biochrom) to kill extracellular bacteria. Following additional washes with PBS, cells were lysed as described above. To evaluate numbers of adherent or invasive bacteria, *C. jejuni* from respective lysates were serially diluted and CFU counted on MHA after 48 h incubation at 37 ℃ under microaerobic conditions. Adhesion and invasion index were calculated as percentage of the inoculum, respectively. Each experiment was performed using at least three biological replicates considering three technical replicates for each.

### Immunofluorescence detection and microscopy

HT-29/B6 cells were grown on 8-well chamber slides (Sarstedt) to ~ 80–90% confluence and then infected with ::Cj1492c, ::Cj1507c and the wild type (MOI 500) for 3 h and subsequently treated with gentamicin as described above. After incubation, the slides were washed three times with PBS and fixed in 3.7% formaldehyde at room temperature for 15 min. Fixed cells were washed with PBS three times and permeabilised with 0.25% Triton X-100 for 15 min at room temperature followed by three washing steps with PBS. Non-specific binding was blocked with Endogenous Avidin/Biotin blocking kit (Abcam, Cambridge, United Kingdom) according to the manufacturer’s protocol followed by incubation in 1% bovine serum albumin (Sigma-Aldrich) in PBST (PBS + 1% Tween 20) for 1 h at room temperature. Cells were subsequently incubated with 1:1000 diluted primary antibody for *C. jejuni* (Biotin-rabbit polyclonal, Abcam) in PBST supplemented with 1% BSA in a humidified chamber overnight at 4 ℃. After three washes with PBS for 5 min, primary antibody was visualised using Alexa Fluor 488 conjugated streptavidin (1:400 diluted) (Thermo Fisher Scientific) following the method previously described with slight modifications [21]. HT-29/B6 cell membranes were subsequently stained with wheat germ agglutinin conjugate WGA-CF^®^594 (Biotium, Fremont, US) at a concentration of 5 µg/ml in Hank’s Balanced Salt Solution (HBSS; Lonza) for 10 min at room temperature, followed by washing twice in PBS. Nuclei were stained with DAPI (200 ng/ml) (Sigma) by 15 min incubation at room temperature. After two washes with PBS, the slides were mounted with 50% glycerol in PBS before fluorescence microscopy using a Leica DMI6000 (Leica, Wetzlar, Germany). The internalisation level of tested strains was calculated as a ratio of invaded *C. jejuni* and infected host cells, which were automatically counted by the ImageJ software as previously described [27]. Briefly, we used ImageJ to outline interested signals by thresholding and then identified particles with a specific size range. Afterwards, the particle counting was automatically measured by the Region of Interest (RoI) manager.

### Cell viability determination

To study effects of *C. jejuni* on HT-29/B6 viability, Calcein-acetoxymethyl/Hoechst staining was performed. Calcein-acetoxymethyl (Calcein-AM; Biotium) has already been described as a green fluorescent indicator of viable cells in cytotoxicity assays [29, 30]. Hoechst 33342 (Thermo Fisher Scientific) (hereinafter referred to as Hoechst) is used as a blue fluorescent marker of the nuclei of all cells [53]. Viability of HT-29/B6 cells was investigated in 96 well plates (Sarstedt). After 7 days differentiation, HT-29/B6 with ~ 80–90% confluence were infected with wild type, ::Cj1492c and ::Cj1507c for 5 h and 24 h as described before. Infected monolayers were washed 3 times with PBS and incubated with 0.4 µM Calcein-AM at 37 ℃ with 5% CO_2_ for 30 min. 5 µg/ml Hoechst was added to each well and incubated 15 min at room temperature followed by two washes with PBS. Fluorescence micrographs were subsequently captured with a Leica DMI6000 (Leica). The relative viability of host cells was calculated as percentage of the number of viable cells compared to the total cell numbers automatically determined using the ImageJ software as described above [27]. Presented results are calculated from three individual assays.

### RNA isolation and RT-qPCR

For mRNA expression analysis, in vitro infection was performed as described above and HT-29/B6 samples were taken at 1, 5 and 24 h p.i. considering respective negative controls (non-infected cells). For this, cell monolayers were washed three times with PBS and lysed directly in each well of 8-well plates by addition of cell lysis buffer (Roboklon, Berlin, Germany). Total RNA was isolated with Universal RNA/miRNA Purification Kit (Roboklon) according to the manufacturer’s protocol. The RNA quality and quantity was controlled using the Agilent 2100 Bioanalyzer with the RNA Nano Chips (Agilent, Waldbronn, Germany) and the DS-11 Spectrophotometer/Fluorometer (DeNovix Inc., Wilmington, USA), respectively. Relative expression of mRNA coding for IL-6 and IL-8 was quantified by RT-qPCR as described earlier [54]. For normalisation of gene expression, ACTB and B2M transcripts were used as reference genes and relative gene expression was calculated as described earlier [54] by the ∆∆Ct method [55]. Experiments were performed in triplicate. Primers used in this study are listed in Table 1.

## Data Availability

All data generated or analysed during this study are included in this published article.
